# Jujuboside A extends healthspan and enhances resistance of oxidative stress in *Caenorhabditis elegans* via the transcription factor DAF-16/FOXO3A and SKN-1/Nrf2

**DOI:** 10.3389/fphar.2026.1816985

**Published:** 2026-06-23

**Authors:** Yunliang Cao, Shuangjie Ren, Xiaocong Li, Xin Xiao, Yi Xiao

**Affiliations:** 1 Department of Oncology, The Second Affiliated Hospital of Zunyi Medical University, Zunyi, China; 2 The Key Lab of Guizhou Provincial Department of Education for Medical Prevention and Treatment of Tumor, Zunyi Medical University, Zunyi, Guizhou, China; 3 Institute of life sciences, Zunyi Medical University, Zunyi, Guizhou, China; 4 College of Basic Medicine, Zunyi Medical University, Zunyi, Guizhou, China; 5 The First People’s Hospital of Zunyi, The Third Affiliated Hospital of Zunyi Medical University, Zunyi, Guizhou, China

**Keywords:** *Caenorhabditis elegans*, DAF-16/FOXO3A, healthspan, jujuboside A, oxidative stress, SKN-1/Nrf2

## Abstract

Jujuboside A (JA) is a biologically active saponin primarily extracted from sour jujube seeds. Existing studies have demonstrated that JA plays significant roles in anti-inflammatory, antioxidant, neuroprotective, and metabolic regulation. However, its mechanisms in lifespan extension and antioxidation remain to be further elucidated. In this study, we found that JA extended the lifespan of *Caenorhabditis elegans* in dose-dependent manner. JA increased the locomotion and decreased the aging pigment but did not affect the reproductive performance in *C. elegans*. Furthermore, by screening the signaling pathway, we showed that JA extends the healthspan in a manner dependent on the transcription factors DAF-16/FOXO3A and SKN-1/Nrf2. In addition, we found that JA enhanced the mRNA levels of DAF-16 target genes, such as *sod-3*, *hsp-12.6*, *dod-3*, *lys-7*, and *thn-2* and SKN-1 target genes, such as *gst-4*, *gst-10*, and *gcs-1*. Meanwhile, JA increased the expression of *sod-3::GFP* and *gst-4::GFP*. Moreover, we found that JA also enhanced oxidative stress resistance in a manner dependent on the transcription factors DAF-16/FOXO3A and SKN-1/Nrf2. In summary, our study demonstrates that JA extends healthspan and enhances the resistance of oxidative stress in *C. elegans* via transcription factor DAF-16/FOXO3A and SKN-1/Nrf2.

## Introduction

Aging is a process during which physiological and psychological functions gradually decline with age, characterized by the degradation of tissue and cellular integrity that eventually leads to organ failure (Zhang Y. et al., 2025; Zhang H. et al., 2025). This process is closely associated with an increased incidence of a variety of age-related diseases, including type 2 diabetes, cancer, hypertension, neurodegeneration diseases (Xiao et al., 2022a; Xiao et al., 2023a). As the global population undergoes an unprecedented demographic shift toward advanced age, the primary goal of anti-aging research is to identify chemical or pharmacological interventions capable of extending healthspan and reducing the risk of age-related diseases (Xiao et al., 2024a). Oxidative stress refers to the imbalance between the production of reactive oxygen species (ROS) and the capacity of antioxidants to neutralize them. While low levels of ROS are essential for normal cellular signaling, excessive ROS can inflict damage on DNA, proteins, and lipids, consequently accelerating the aging process and triggering various diseases (Liu et al., 2022; Xiao et al., 2025a). Thus, antioxidant interventions may counteract aging, and pharmacological strategies targeting antioxidant pathways have emerged as a research hotspot.

Jujuboside A (JA), a triterpenoid saponin extracted from the seeds of Ziziphus jujuba var. spinosa, has attracted widespread attention due to its diverse pharmacological activities, particularly its significant effects on the nervous, metabolic, and cardiovascular systems. It exhibits notable sedative, hypnotic, antioxidant, anti-epileptic, and anti-tumor activities, as well as beneficial effects in models of Alzheimer’s disease (Zhou et al., 2026). However, whether JA can extend animal lifespan and the underlying mechanisms remain largely unexplored. Some studies suggest that JA may inhibit oxidative stress-induced damage by upregulating IDO expression and enhancing the immunomodulatory capacity of human umbilical cord mesenchymal stem cells via the YY1/CYP2E1 signaling pathway (Zhang et al., 2024). Additionally, by ameliorating hepatic lipid accumulation, inflammation, and oxidative stress, it exerts protective effects that counteract oxidative stress and inflammatory responses induced by traumatic epilepsy (Lu et al., 2022). Clearly, further research is needed to elucidate the mechanisms underlying its antioxidant properties.

*Caenorhabditis elegans* has been firmly established as a premier model organism for oxidative stress and anti-aging research due to its numerous advantages, including its high homology with mammals, conserved oxidative stress response pathways, and the presence of aging-related loss-of-function mutations (Xiao et al., 2022b; Xiao et al., 2025b). Based on this superior model, researchers have proposed several signaling pathways associated with aging and oxidative stress, including the insulin/IGF-1 signaling pathway (IIS) (Xiao et al., 2024a), the TOR signaling pathway (Xiao et al., 2022a), SKN-1/Nrf2 (Liu et al., 2022), the mitochondrial unfolded protein response (UPR^mt^) (Xiong et al., 2025; Cui et al., 2025), the hypoxia-inducible factor (Zhang et al., 2009), and the endoplasmic reticulum unfolded protein response (UPR^er^) (Liu et al., 2025a; Xiao et al., 2024b). Among the key transcriptional effectors that integrate signals from these upstream pathways to orchestrate cellular defense and longevity programs are DAF-16/FOXO3A and SKN-1/Nrf2. DAF-16, the sole *Caenorhabditis elegans* ortholog of the mammalian FOXO family, serves as a master regulator of stress resistance, metabolism, and lifespan determination (Ewald et al., 2018; Liu et al., 2025b). Similarly, SKN-1 is a transcription factor in *C. elegans*, with functions analogous to the mammalian Nrf2, responsible for coordinating oxidative stress defense, detoxification, development, and lifespan regulation (Liu et al., 2022). Its activity is rigorously controlled by various signaling pathways and post-translational modifications, enabling the organism to adapt to environmental and metabolic challenges.

Based on the structural features of Jujuboside A as a bioactive triterpenoid saponin with documented antioxidant properties and the evolutionary conservation of DAF-16/FOXO3A and SKN-1/Nrf2 as central orchestrators of longevity and oxidative stress resistance, we hypothesize that Jujuboside A extends lifespan and enhances oxidative stress resistance in *C. elegans* primarily in a manner dependent on the transcription factors DAF-16/FOXO3A and SKN-1/Nrf2. The experimental design of this study was specifically structured to test this mechanistic hypothesis.

## Results

### Jujuboside A promotes lifespan and healthspan in *Caenorhabditis elegans*

**FIGURE 1:**
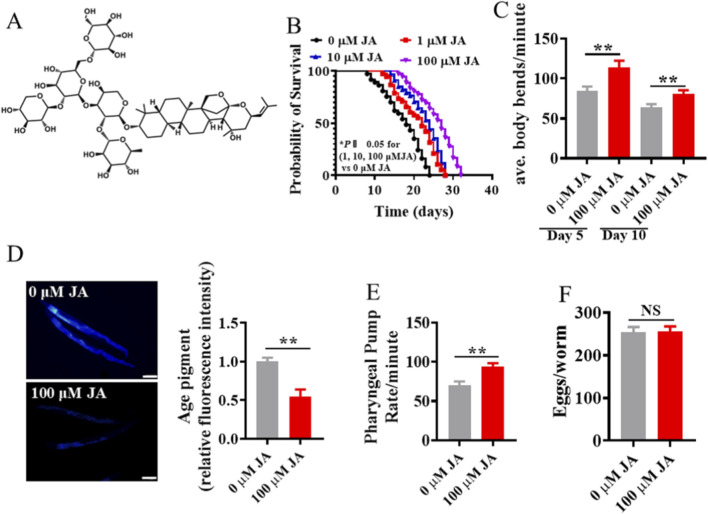
Jujuboside A promotes lifespan and healthspan in *C. elegans*. **(A)** Illustration of the chemical structure of Jujuboside A (JA). **(B)** Survival curves for N2 hermaphrodite worms that were exposed to increasing concentrations of JA (*P < 0.05; Chi-square = 62.78; Median lifespan: Control, 18; 1 μM JA, 22; 10 μM JA, 24; 100 μM JA, 27; log-rank test). **(C)** Locomotion was evaluated by counting the number of body bends per minute (n ≥ 10; **P < 0.01, unpaired t-test). **(D)** Analysis of aging pigments is presented by quantifying lipofuscin fluorescence normalized to autofluorescence (n ≥ 20; **P < 0.01, unpaired t-test). **(E)** Pharyngeal pumping rates of control and JA treatment group are counted as the number of pumps per min. (n ≥ 10; **P < 0.01, unpaired t-test). **(F)** fertility was measured by determining the number of eggs produced per worm (n ≥ 10; **P < 0.01, unpaired t-test). Error bars represent the mean ± SEM from three independent biological replicates. JA is the abbreviation for Jujuboside A, and NS indicates results that are not statistically significant. *P < 0.05 and **P < 0.01 indicate statistically significant differences.

Figure 1A shows the chemical structure of Jujuboside A (JA). To investigate the effects of JA on the lifespan of *Caenorhabditis elegans*, we measured the lifespan of the nematodes exposed to different concentrations of JA (0, 1, 10, and 100 μM). Concentrations of JA (1, 10, 100 μM) were selected based on preliminary toxicity assays and previous studies demonstrating bioactivity without causing adverse developmental effects (Hao et al., 2023). The results demonstrated that JA extended the lifespan of *C. elegans*, with the lifespan extension becoming more pronounced as the concentration of JA increased, and the most significant effect observed at 100 μM (Figure 1B). As the nematodes aged, they exhibited muscle degeneration and a decline in locomotion, which are hallmark indicators of aging and health in *C. elegans* (Xiao et al., 2023b). Our findings indicated that under the influence of 100 μM JA, the age pigments of *C. elegans* were significantly reduced while their body bending and pharyngeal pumping increased markedly, suggesting that JA is beneficial to the health of the nematodes (Figures 1C–E). However, we did not observe any reduction in the reproductive capacity between worms treated with 100 μM JA and the untreated group (Figure 1F). These results demonstrate that Jujuboside A promotes both lifespan and healthspan in *Caenorhabditis elegans*.

### Jujuboside A requires DAF-16 and SKN-1 to extend lifespan

**FIGURE 2:**
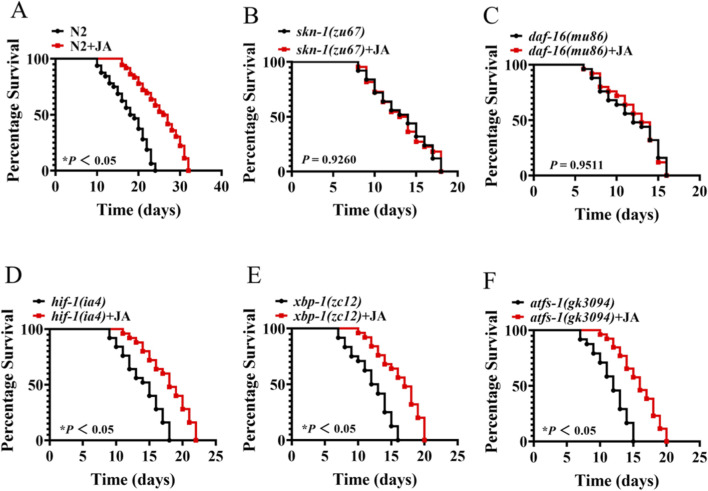
Jujuboside A requires DAF-16 and SKN-1 to extend lifespan. **(A)** Survival of N2 treated with 100 μM JA and the untreated controls. (*P < 0.05; Chi-square = 33.16; Hazard Ratio (95% CI): 0.31 (0.18-0.55); Median lifespan: Control, 18.5; JA, 26.5; log-rank test). **(B)** Survival of *skn-1(zu67)* treated with 100 μM JA and the untreated controls. (*P* = 0.9260; Chi-square = 0.0086; Hazard Ratio (95% CI): 0.98 (0.55-1.73); Median lifespan: Control, 14; JA, 13.5; log-rank test). **(C)** Survival of *daf-16(mu86)* treated with 100 μM JA and the untreated controls (*P* = 0.9511; Chi-square = 0.0038; Hazard Ratio (95% CI): 0.98 (0.57-1.71); Median lifespan: Control, 12; JA, 13; log-rank test). **(D)** Survival of *hif-1(ia4)* treated with 100 μM JA and the untreated controls. (*P < 0.05; Chi-square = 16.32; Hazard Ratio (95% CI): 0.39 (0.21-0.72); Median lifespan: Control, 15; JA, 18; log-rank test). **(E)** Survival of *xbp-1(zc12)* treated with 100 μM JA and the untreated controls. (*P < 0.05; Chi-square = 20.34; Hazard Ratio (95% CI): 0.35 (0.18-0.66); Median lifespan: Control, 15; JA, 18; log-rank test). **(F)** Survival of *atfs-1(gk3094)* treated with 100 μM JA and the untreated controls. (*P < 0.05; Chi-square = 22.78; Hazard Ratio (95% CI): 0.33 (0.17-0.64); Median lifespan: Control, 12; JA, 16; log-rank test).

To investigate the molecular mechanism by which Jujuboside A extended lifespan, we screened several key transcription factors involved in longevity regulation that regulated longevity in *Caenorhabditis elegans*, such as DAF-16/FOXO3A (Liu et al., 2025b), SKN-1/Nrf2 (Liu et al., 2022), HIF-1 (Chen et al., 2009), XBP-1 (Zhou et al., 2025), ATFS-1 (Xiao et al., 2024c; Xiao et al., 2025c). We found that 100 μM JA did not extend the lifespan of *skn-1(zu67)* and *daf-16(mu86)* mutant animals (Figures 2A–C). However, 100 μM JA extended the lifespan of *hif-1(ia4)*, *xbp-1(zc12)*, and *atfs-1(gk3094)* mutant worms (Figures 2D–F). These results indicate that the lifespan extension induced by Jujuboside A is dependent on the presence of functional DAF-16/FOXO3A and SKN-1/Nrf2.

### Jujuboside A extends healthspan via the transcription factor SKN-1/Nrf2 in *Caenorhabditis elegans*

**FIGURE 3:**
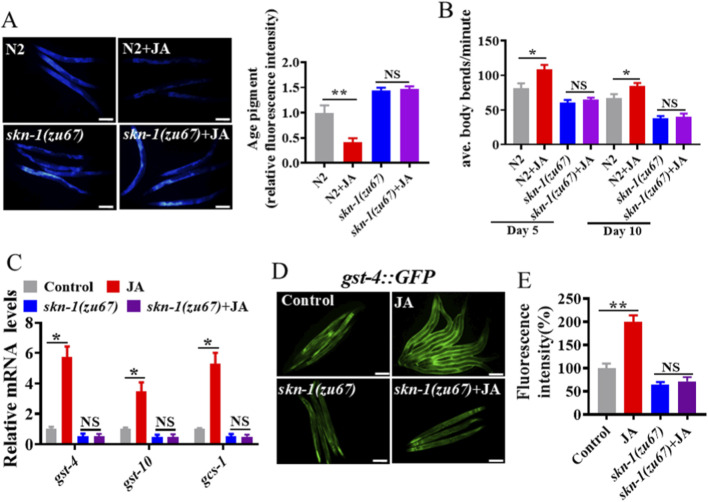
Jujuboside A extends healthspan by the transcription factor SKN-1/Nrf2 in *C. elegans*. **(A)** By normalizing lipofuscin fluorescence to autofluorescence for quantitative analysis, the study presents the findings on aging pigments by comparing N2 worms treated with 100 μM JA to untreated controls, as well as *skn-1(zu67)* mutants treated with 100 μM JA to their corresponding untreated controls. (n ≥ 20; **P < 0.01, unpaired t-test). **(B)** The locomotor ability was evaluated by quantifying the number of body bends per minute to compare the performance of N2 worms treated with 100 μM JA against their untreated controls, as well as that of *skn-1(zu67)* mutants treated with 100 μM JA against their corresponding untreated controls. (n ≥ 10; *P < 0.05, unpaired t-test). **(C)** Quantitative real-time PCR was performed to compare the mRNA levels of three SKN-1 target genes (*gst-4*, *gst-10* and *gcs-1*) in N2 worms treated with 100 μM JA with those in untreated control nematodes, and in *skn-1(zu67)* mutants treated with 100 μM JA with those in their corresponding untreated control worms. These results are mean ± SEM of three independent experiments performed in triplicate. (*P < 0.05, one-way ANOVA). **(D)** Images of the *gst-4::GFP* transgenic strain are shown to compare *gst-4* expression in N2 worms treated with 100 μM JA with that in untreated controls, as well as in *skn-1(zu67)* mutants treated with 100 μM JA with that in their corresponding untreated controls. **(E)** Results of the quantitative analysis of fluorescence intensity. (n ≥ 20). Scale bars: 100 μm. These results are mean ± SEM of three independent experiments performed in triplicate. (**P < 0.01, one-way ANOVA). Error bars represent the mean ± SEM from three independent biological replicates. JA is the abbreviation for Jujuboside A, and NS indicates results that are not statistically significant. *P < 0.05 and **P < 0.01 indicate statistically significant differences.

SKN-1/Nrf mediates the regulation of stress adaptation and lifespan-related genes in *Caenorhabditis elegans*, and thus its activation level is closely associated with healthspan (Liu et al., 2022). We found that 100 μM JA reduced the age pigments and increased body bends in N2 worms, but did not change these phenotypes in *skn-1(zu67)* mutant worms (Figures 3A,B), suggesting that JA may confer health benefits via the SKN-1/Nrf2 pathway. To determine whether JA requires the transcription factor SKN-1/Nrf2, we subsequently examined the expression of SKN-1 target genes, such as *gst-4*, *gst-10*, and *gcs-1* (Liu et al., 2022). Quantitative real-time PCR analysis revealed that 100 μM JA increased the mRNA levels of SKN-1 target genes compared to control group (Figure 3C). However, 100 μM JA failed to enhance the expression of SKN-1 target genes in *skn-1(zu67)* mutants (Figure 3C). Furthermore, we tested *gst-4* expression by using transgenic worms expressing *gst-4::GFP*. We observed elevated GFP levels in the 100 μM JA-treated group, but not in the *skn-1(zu67)* mutant (Figures 3D,E). These results indicate that jujuboside A promotes healthspan by the transcription factor SKN-1/Nrf2.

### Jujuboside A extends healthspan via the transcription factor DAF-16/FOXO3A in *Caenorhabditis elegans*

**FIGURE 4:**
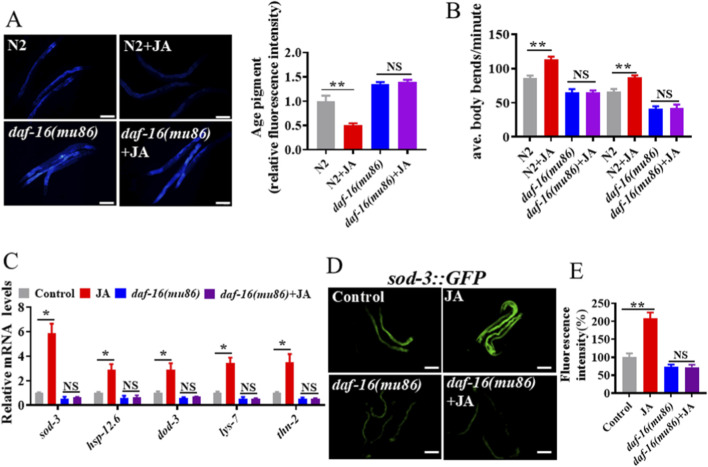
Jujuboside A extends healthspan by the transcription factor DAF-16/FOXO3A in *C. elegans*. **(A)** By normalizing lipofuscin fluorescence to autofluorescence for quantitative analysis, the study presents the findings on aging pigments by comparing N2 worms treated with 100 μM JA to untreated controls, as well as *daf-16(mu86)* mutants treated with 100 μM JA to their corresponding untreated controls. (n ≥ 20; **P < 0.01, unpaired t-test). **(B)** The locomotor ability was evaluated by quantifying the number of body bends per minute to compare the performance of N2 worms treated with 100 μM JA against their untreated controls, as well as that of *daf-16(mu86)* mutants treated with 100 μM JA against their corresponding untreated controls. (n ≥ 10; **P < 0.01, unpaired t-test). **(C)** Quantitative real-time PCR was performed to compare the mRNA levels of three DAF-16 target genes (*sod-3*, *hsp-12.6*, *dod-3*, *lys-7* and *thn-2*) in N2 worms treated with 100 μM JA with those in untreated control nematodes, and in *daf-16(mu86)* mutants treated with 100 μM JA with those in their corresponding untreated control worms. These results are mean ± SEM of three independent experiments performed in triplicate. (*P < 0.05, one-way ANOVA) **(D)** Images of the *sod-3::GFP* transgenic strain are shown to compare *gst-4* expression in N2 worms treated with 100 μM JA with that in untreated controls, as well as in *daf-16(mu86)* mutants treated with 100 μM JA with that in their corresponding untreated controls. **(E)** Results of the quantitative analysis of fluorescence intensity. (n ≥ 20). Scale bars: 100 μm. These results are mean ± SEM of three independent experiments performed in triplicate. (**P < 0.01, one-way ANOVA). Error bars represent the mean ± SEM from three independent biological replicates. JA is the abbreviation for Jujuboside A, and NS indicates results that are not statistically significant. *P < 0.05 and **P < 0.01 indicate statistically significant differences.

The DAF-16/FOXO3A transcription factor plays a pivotal role in aging and longevity (Zhang Y. et al., 2025). Our experiments demonstrated that treatment with 100 μM JA reduced the age pigments and increased body bends in N2 worms, but did not change these phenotypes in *daf-16(mu86)* mutant worms (Figures 4A,B), suggesting that JA may confer health benefits via the DAF-16/FOXO3A pathway. To further assess whether JA requires the DAF-16/FOXO3A transcriptional complex, we examined the expression of several of its target genes, including *sod-3*, *hsp-12.6*, *dod-3*, *lys-7*, and *thn-2* (Zhang H. et al., 2025). Quantitative real-time PCR analyses revealed that 100 μM JA significantly elevated the mRNA levels of these genes in control worms (Figure 4C). However, no such induction was observed in *daf-16(mu86)* mutants. In addition, transgenic worms expressing *sod-3::GFP* exhibited increased GFP fluorescence following 100 μM JA treatment, an effect that was absent in the *daf-16(mu86)* mutant background (Figures 4D,E). Collectively, these results indicate that jujuboside A promotes healthspan by the DAF-16/FOXO3A transcription factor.

### Jujuboside A enhances oxidative stress resistance through DAF-16/FOXO3A and SKN-1/Nrf2

**FIGURE 5:**
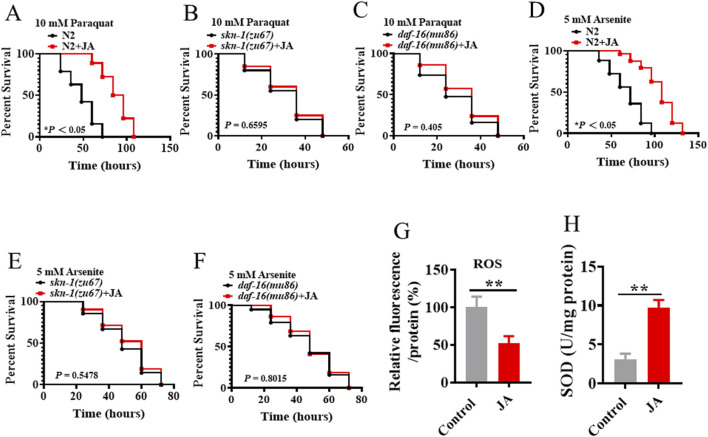
Jujuboside A enhances the resistance of oxidative stress via transcription factor DAF-16/FOXO3A and SKN-1/Nrf2. **(A)** The survival of N2 worms treated with 100 μM JA was compared to that of the untreated control group by exposing them to 10 mM paraquat to generate survival curves. (*P < 0.05; Chi-square = 28.30; Hazard Ratio (95% CI): 0.27 (0.12-0.58); Median lifespan: Control, 48; JA, 90; log-rank test). **(B)** Survival curves were obtained by exposing worms to 10 mM paraquat, comparing the survival of *skn-1(zu67)* mutants treated with 100 μM JA to that of untreated control groups. (*P* = 0.6595; Chi-square = 0.19; Hazard Ratio (95% CI): 0.91 (0.49-1.69); Median lifespan: Control, 36; JA, 36; log-rank test). **(C)** Survival curves were obtained by exposing worms to 10 mM paraquat, comparing the survival of *daf-16(mu86)* mutants treated with 100 μM JA to that of untreated control groups. (*P* = 0.405; Chi-square = 0.10; Hazard Ratio (95% CI): 0.83 (0.45-1.56); Median lifespan: Control, 24; JA, 36; log-rank test). **(D)** The survival of N2 worms treated with 100 μM JA was compared to that of the untreated control group by exposing them to 5 mM arsenite to generate survival curves. (*P < 0.05; Chi-square = 30.02; Hazard Ratio (95% CI): 0.30 (0.15-0.57); Median lifespan: Control, 72; JA, 108; log-rank test). **(E)** Survival curves were obtained by exposing worms to 5 mM arsenite, comparing the survival of *skn-1(zu67)* mutants treated with 100 μM JA to that of untreated control groups. (*P* = 0.5478; Chi-square = 0.36; Hazard Ratio (95% CI): 0.87 (0.48-1.60); Median lifespan: Control, 48; JA, 60; log-rank test). **(F)** Survival curves were obtained by exposing worms to 5 mM arsenite, comparing the survival of *daf-16(mu86)* mutants treated with 100 μM JA to that of untreated control groups. (*P* = 0.8015; Chi-square = 0.063; Hazard Ratio (95% CI): 0.94 (0.51-1.75); Median lifespan: Control, 48; JA, 48; log-rank test). **(G)** Quantitative analysis of ROS levels in nematodes with and without JA treatment. (**P < 0.01, unpaired t-test). **(H)** Detection of SOD activity in JA-treated and untreated worms. (**P < 0.01, unpaired t-test). Error bars represent the mean ± SD from three independent biological replicates. JA is the abbreviation for Jujuboside A, and NS indicates results that are not statistically significant. *P < 0.05 and **P < 0.01 indicate statistically significant differences.

*Caenorhabditis elegans* serves as a widely employed model organism for studying oxidative stress due to its genetic manipulability, short lifespan, and conservation of key stress response pathways with humans (Xiao et al., 2025b). Previous studies have demonstrated that sensitivity to oxidative stress can be quantified by measuring survival rates following exposure to paraquat and arsenite (Zhu et al., 2022). Our findings indicated that exposure of *C. elegans* to 10 mM paraquat and 5 mM arsenite, respectively, 100 μM JA extended the survival rate of N2 worms. However, 100 μM JA did not prolong the survival rates of *skn-1(zu67)* and *daf-16(mu86)* mutant animals (Figures 5A–F). The oxidative stress theory of aging holds that reactive oxygen species (ROS) generated by normal metabolism accumulate and damage intracellular macromolecules, disrupt cellular components, and thereby shorten the lifespan of *Caenorhabditis elegans* (Liu et al., 2022). We found that JA significantly reduced the ROS level in nematodes (Figure 5G). Meanwhile, studies have shown that superoxide dismutase (SOD) plays a crucial role in antioxidant defense (Fridovich, 1978). Detection of SOD activity revealed that JA significantly upregulated the SOD level in nematodes (Figure 5H). These findings demonstrate that JA enhances organismal resistance to oxidative stress through DAF-16/FOXO3A and SKN-1/Nrf2.

## Discussion

The present study was designed to investigate whether Jujuboside A (JA), a triterpenoid saponin with documented cytoprotective properties, extends organismal lifespan and enhances oxidative stress resistance through the evolutionarily conserved transcription factors DAF-16/FOXO and SKN-1/Nrf2. Our results demonstrate that JA significantly prolongs both lifespan and healthspan in *Caenorhabditis elegans*, an effect strictly contingent upon the presence of functional DAF-16 and SKN-1, as evidenced by the complete abrogation of these benefits in corresponding null mutant strains. Although the pharmacological profile of JA has been extensively characterized in mammalian disease models—particularly its capacity to ameliorate insomnia, neurodegeneration, and metabolic dysfunction via modulation of GABAergic tone (Wang M. et al., 2025; Wang W. et al., 2025), mitochondrial integrity (Zhang Z. et al., 2025), and specific kinase cascades (Chen et al., 2022)—its mechanistic role in the fundamental biology of aging remains largely unexplored. Prior investigations have predominantly focused on disease-specific endpoints within pathological challenge paradigms, an approach that may inadvertently obscure the subtler homeostatic regulators governing normative aging. Departing from this paradigm, our study reveals a conserved pro-longevity mechanism operating at the organismal level, thereby extending the known bioactivity of JA beyond targeted therapeutic intervention and establishing it as a *bona fide* geroprotective agent. Critically, our findings both align with and substantively expand upon existing mechanistic frameworks. Consistent with previous reports demonstrating that JA mitigates oxidative injury and suppresses pro-inflammatory cascades in mammalian tissues through Nrf2-mediated transcriptional activation of phase II detoxification enzymes, we confirm the essential contribution of the SKN-1/Nrf2 axis to JA-induced stress tolerance and the upregulation of canonical targets such as *gst-10*, *gst-4* and *gcs-1*. This cross-phylum conservation underscores the foundational nature of the Nrf2 pathway as a core effector. A key point of divergence from the extant literature, however, lies in our identification of DAF-16/FOXO as an equally indispensable mediator of JA-dependent longevity. Previous work in mammalian systems has largely ascribed the protective effects of JA to the modulation of upstream kinases—including the PI3K/Akt/mTOR cascade (Han et al., 2016), p38/ERK1/2 signaling (Lu et al., 2022), or the Axl/HSP90/PPARγ axis (Zhang et al., 2018)—without directly interrogating the functional engagement of FOXO transcription factors in the absence of overt pathology. This discrepancy likely stems from fundamental differences in experimental design; acute stress or disease-centric models may afford only a limited therapeutic window for observing FOXO-dependent transcriptional outputs, particularly when concurrent survival pathways are co-activated or disease-specific targets predominate. By contrast, the *C. elegans* model permits precise genetic dissection of longevity pathways under basal, unstressed conditions throughout the entire adult lifespan, thereby unveiling a critical role for DAF-16 that might otherwise remain cryptic in short-term or injury-focused assays. Consequently, the unique contribution of this study lies not only in confirming the antioxidant properties of JA, but also in extending its scope of action at the mechanistic level through the combined influence of the transcription factors DAF-16/FOXO3A and SKN-1/Nrf2, thereby establishing a dual-factor dependency for JA. While many natural compounds modulate these pathways individually, our data suggest a ‘dual-factor dependency’ for JA. However, it remains to be elucidated whether JA regulates these pathways through a distinct upstream mechanism or whether simultaneous engagement of DAF-16 and SKN-1 produces a unique biological outcome beyond that described for other compounds. This functional interdependence between two master regulators of aging provides a superior explanatory framework for the pleiotropic benefits of JA observed across diverse physiological contexts, distinguishing our findings from prior investigations that have focused predominantly on singular pathway outputs.

Several inherent limitations warrant careful consideration when interpreting the scope and mechanistic depth of these findings. First and foremost, while our genetic epistasis experiments unequivocally establish DAF-16 and SKN-1 as indispensable downstream effectors of JA-mediated longevity and stress resistance, the current dataset supports a model of functional pathway dependency rather than direct biochemical activation. The precise molecular targets of JA and the immediate upstream signaling events that culminate in the transcriptional engagement of these factors remain undefined. Critically, the absence of direct evidence—such as quantitative nuclear localization assays or biochemical binding data—precludes the formulation of a definitive mechanism-of-action model. We acknowledge that although the enhanced expression of DAF-16 and SKN-1 target genes and the complete abrogation of JA effects in null mutants confirm the essential requirement for these transcription factors, such genetic necessity does not distinguish between direct activation, indirect signaling convergence, or permissive downstream involvement. We have partially mitigated this interpretive limitation by employing complementary transcriptional reporter assays to validate functional pathway output; however, future studies employing high-resolution live-cell imaging of DAF-16::GFP and SKN-1::GFP subcellular localization dynamics, combined with biochemical approaches to identify direct JA binding partners, will be essential to resolve the precise mechanism and the signaling hierarchy linking JA exposure to transcription factor engagement. Second, this investigation is confined to a single invertebrate model system. Although the remarkable evolutionary conservation of the transcription factor DAF-16/FOXO3A and SKN-1/Nrf2 across metazoans provides a compelling rationale for extrapolation, the translational relevance of geroprotective compounds identified in nematodes does not universally extend to vertebrate physiology. Confirmatory validation in vertebrate models therefore remains a necessary prerequisite for evaluating the broader applicability of these findings.

**FIGURE 6:**
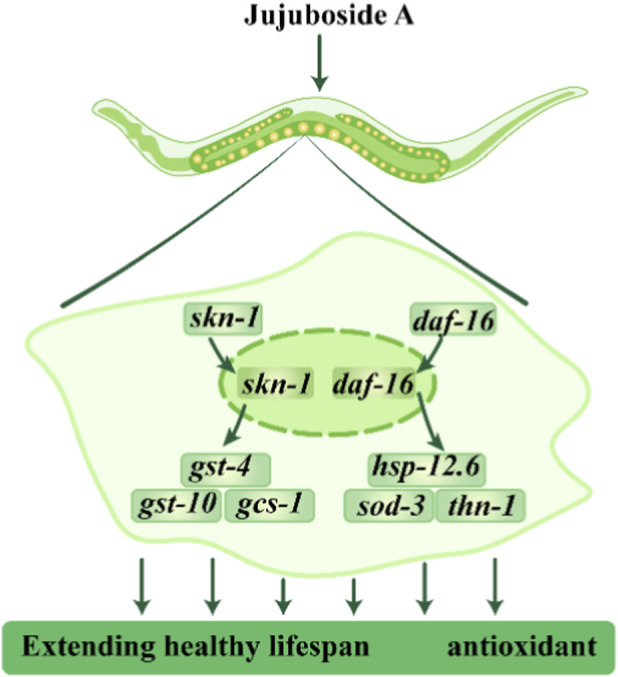
The mechanisms of Jujuboside A-mediated healthspan and antioxidant.

In summary, this study identifies Jujuboside A extends healthspan and enhances resistance of oxidative stress in *Caenorhabditis elegans* via the transcription factor DAF-16/FOXO3A and SKN-1/Nrf2 (Figure 6). Beyond its immediate contribution to the pharmacological characterization of this phytochemical, the unique advance of this work lies in establishing dual-factor dependency for JA—thereby moving beyond the well-documented single-axis modulation of Nrf2 to delineate a more integrative mechanism of systemic stress resistance. Looking forward, future investigations should prioritize identifying the direct molecular targets of JA and the specific upstream signaling events governing DAF-16 and SKN-1 activation. Furthermore, validating these effects in vertebrate models will be essential to determine the translational potential of JA as a geroprotective intervention.

## Materials and methods

### Chemicals

Jujuboside A (JA) was purchased from Baoji Herbest Bio-Tech Co., Ltd (CAS: 491-80-5, Baoji City, Shanxi Province, China) and prepared as a 100 mM stock solution using dimethyl sulfoxide (DMSO) as the solvent and stored at −20 °C after aliquoting.

### Worm strains and cultivation

Worms are maintained and propagated under standard conditions (Xiao et al., 2020; Xiao et al., 2025d). The following *Caenorhabditis elegans* strains were provided by the *Caenorhabditis elegans* Genetics Center (CGC) at the University of Minnesota, USA. N2 Bristol wild-type, SJ17 *xbp-1(zc12)*, VC3201 *atfs-1(gk3094)*, EU31 *skn-1(zu67)*, CF1038 *daf-16(mu86)*, XZ2056 *hif-1(ia4)*, CF1553 (*sod-3p::GFP*), CL2166 (*gst-4p::GFP*).

### Lifespan assays

All lifespan analyses were conducted at 20 °C according to standard protocols. In brief, synchronized L1 to L2 larvae were placed on NGM plates and allowed to mature at 20 °C (Xiao et al., 2023b). Approximately 100 L4-stage nematodes were manually transferred to fresh assay plates containing Jujuboside A (0, 1, 10, or 100 μM). During the first 10-14 days, worms were transferred daily and thereafter every other day. Worms that did not respond to mild stimuli were scored as dead. Animals that climbed off the plates or exhibited unnatural death, particularly those dying from internal hatching, were censored (Xiao et al., 2023b). Three plates were used in each trial, and all experiments were independently performed in triplicate. Experiments were performed in a randomized manner, and investigators were blinded to the treatment groups during data analysis where applicable.

### Locomotion assays

Starting from the L4 larval stage, nematodes were synchronously treated either with or without 100 μM Jujuboside A. On the 5th and 10th day of adulthood, 30 individuals from both the control and experimental plates were selected to measure body curvature in liquid. In short, the worms were placed in 20 μL of M9 buffer on a slide and imaged using a Zeiss Imager M2 microscope. Body curvature was calculated by reviewing each frame of a 60-s video (Xiao et al., 2023b). Each experiment tested approximately 30 animals per plate, and all assays were independently conducted three times. Experiments were performed in a randomized manner, and investigators were blinded to the treatment groups during data analysis where applicable.

### Age pigment fluorescence detection

Starting from the L4 larval stage, worms were synchronized and treated for 10 days with or without 100 μM Jujuboside A. The worms were mounted on agarose pads attached to glass slides and imaged using a Zeiss Imager M2 microscope. Fluorescence intensity was measured with ImageJ (Xiao et al., 2023b). Approximately 30 animals per plate were tested in each experiment, and all experiments were performed independently three times. Experiments were performed in a randomized manner, and investigators were blinded to the treatment groups during data analysis where applicable.

### Fertility assay

Starting at the L4 larval stage, worms were synchronously treated with or without 100 μM Jujuboside A. The worms were transferred to individual seeded plates and subsequently moved to fresh plates each day during the reproductive period. From stage L4 onwards, the total egg production over the previous 3 days has been recorded. Eggs were manually counted. In each assay, approximately 30 worms per plate were tested, and all experiments were independently conducted three times. Experiments were performed in a randomized manner, and investigators were blinded to the treatment groups during data analysis where applicable.

### Quantitative real-time PCR

From the L4 larval stage onwards, synchronise and treat nematodes for 1 day with or without 100 μM Jujuboside A. Extract total RNA from worms (approximately 2000 individuals) using TRIzol reagent (Invitrogen) (Xiao et al., 2017). Reverse transcribe total RNA samples with SuperScript II (Invitrogen) to generate random primer cDNA. SYBR Premix-Ex Tag™ (Takara, Dalia, China) was employed for QPCR analysis on an Applied Biosystems Prism 7000 Sequence Detection System (Applied Biosystems, Foster City, CA). *pmp-3* served as the internal control (Xiao et al., 2024d; Xiao et al., 2024e). All experiments were independently replicated threefold. The primers were used for this study listed in Supplementary Table S1.

### Fluorescence microscopy

*SOD-3::GFP* and *GST-4::GFP* strains were synchronized and exposed, at the L4 larval stage, either to 100 μM Jujuboside A or left untreated. Fluorescence images were captured using a Carl Zeiss fluorescence microscope (Jena, Germany) equipped with a Zeiss Axiom 2 Plus digital camera at a combined magnification of 400× (40× objective and 10× eyepiece). The fluorescence intensities were then quantified via NIH’s ImageJ software (Liu et al., 2017). In each experiment, approximately 30 animals per plate were examined, with the entire procedure independently repeated three times. Experiments were performed in a randomized manner, and investigators were blinded to the treatment groups during data analysis where applicable.

### Antioxidant experiment

Synchronized L1-L2 larvae were placed on NGM plates and allowed to develop at 20 °C. Approximately 100 L4 nematodes were manually transferred to fresh test plates containing either 100 μM jujuboside A with 10 mM paraquat or 100 μM jujuboside A with 10 mM arsenite (Zhu et al., 2022). Individuals that did not respond to mild stimuli were deemed deceased. Worms that crawled off the plates or exhibited non-natural death (particularly due to internal hatching) were excluded from the analysis. Three plates were used for each experimental group, and all experiments were independently replicated three times. Experiments were performed in a randomized manner, and investigators were blinded to the treatment groups during data analysis where applicable.

### Measurement of ROS

The ROS levels were detected using 2′,7′-dichlorodihydrofluorescein diacetate (DCF-DA) as a fluorescent probe (Liu et al., 2022). Briefly, nematodes were treated with or without 100 μM JA for 24 h. Approximately 1200 worms in each group were collected in M9 buffer and washed three times to eliminate residual bacteria. Subsequently, the worms were homogenized in PBST (PBS containing 1% Tween 20), followed by centrifugation at 12,000 rpm and 4 °C. A total of 100 μL of the supernatant was mixed with an equal volume of 100 mM DCF-DA solution prepared in PBS and incubated at 37 °C for 1 h. The fluorescence intensity was then determined using a Spectra Max M5 microplate reader (Molecular Devices, Sunnyvale, CA) with an excitation wavelength of 488 nm and an emission wavelength of 535 nm. The kinetic fluorescence signals of samples were recorded every 20 min for a total of 2.5 h. Finally, the protein concentration of the supernatant was quantified via the bicinchoninic acid (BCA) assay to normalize the fluorescence intensity of each sample.

### Activities of superoxide dismutases (SOD)

This experiment was conducted with slight modifications to previously established protocols (Hu et al., 2021). Briefly, nematodes were incubated with or without 100 μM JA at 20 °C for 24 h, followed by exposure to 35 °C for 6 h to induce oxidative stress damage. Thereafter, all worms were rinsed three times with M9 buffer and transferred into 2 mL centrifuge tubes, with approximately 1200 individuals per sample. Nematodes were homogenized using an ultrasonic probe, and the supernatant was harvested after centrifugation. The activity of superoxide dismutase (SOD) in each sample was measured strictly according to the manufacturer’s instructions of the commercial assay kit.

### Quantification and statistical analysis

Data are presented as mean ± SEM. Figures were produced using GraphPad Prism 9.0 (GraphPad, San Diego, CA, USA). For all analyses except lifespan evaluation, we applied either an unpaired two-tailed Student's t-test or ANOVA after verifying the data maintained both equal distribution and variance. Survival outcomes were assessed using the log-rank (Mantel-Cox) test, with *P* < 0.05 denoting statistical significance.

## Data Availability

The original contributions presented in the study are included in the article/Supplementary Material, further inquiries can be directed to the corresponding author.

## References

[B1] Chen D. Thomas E. L. Kapahi P. (2009). HIF-1 modulates dietary restriction-mediated lifespan extension via IRE-1 in Caenorhabditis elegans. PLoS Genet. 5, e1000486. 10.1371/journal.pgen.1000486 19461873 PMC2676694

[B2] Chen C. H. Hsu P. C. Hsu S. W. Hong K. T. Chen K. Y. He J. L. (2022). Protective effects of jujubosides on 6-OHDA-Induced neurotoxicity in SH-SY5Y and SK-N-SH cells. Molecules 27, 4106. 10.3390/molecules27134106 35807356 PMC9268520

[B3] Cui Y. Wang R. Li X. Bai G. Xiao Y. (2025). Ginkgolide a enhances the resistance to pathogen infection through mitochondrial unfolded protein response. Cell Mol. Life Sci. 82, 349. 10.1007/s00018-025-05869-5 41055707 PMC12504166

[B4] Ewald C. Y. Castillo-Quan J. I. Blackwell T. K. (2018). Untangling longevity, dauer, and healthspan in Caenorhabditis elegans Insulin/IGF-1-Signalling. Gerontology 64, 96–104. 10.1159/000480504 28934747 PMC5828946

[B5] Fridovich I. (1978). The biology of oxygen radicals. Science 201, 875–880. 10.1126/science.210504 210504

[B6] Han D. Wan C. Liu F. Xu X. Jiang L. Xu J. (2016). Jujuboside A protects H9C2 cells from isoproterenol-induced injury via activating PI3K/Akt/mTOR signaling pathway. Evid. Based Complement. Altern. Med. 2016, 9593716. 10.1155/2016/9593716 27293469 PMC4884826

[B7] Hao K. X. Xie H. Jiang J. G. Wang D. Zhu W. (2023). Semen ziziphus jujube saponins protects HaCaT cells against UV damage and alleviates the aging of Caenorhabditis elegans. ACS Omega 8, 28080–28089. 10.1021/acsomega.3c00433 37576697 PMC10413363

[B8] Hu Q. Liu Z. Guo Y. Lu S. Du H. Cao Y. (2021). Antioxidant capacity of flavonoids from folium artemisiae argyi and the molecular mechanism in Caenorhabditis elegans. J. Ethnopharmacol. 279, 114398. 10.1016/j.jep.2021.114398 34242729

[B9] Liu F. Xiao Y. Ji X. L. Zhang K. Q. Zou C. G. (2017). The cAMP-PKA pathway-mediated fat mobilization is required for cold tolerance in C. elegans. Sci. Rep. 7, 638. 10.1038/s41598-017-00630-w 28377576 PMC5428847

[B10] Liu F. Wang H. Zhu X. Jiang N. Pan F. Song C. (2022). Sanguinarine promotes healthspan and innate immunity through a conserved mechanism of ROS-Mediated PMK-1/SKN-1 activation. iScience 25, 103874. 10.1016/j.isci.2022.103874 35243236 PMC8857505

[B11] Liu F. Wang Q. Xiong J. Wang M. Zhou H. Xiao Y. (2025a). Parental S-adenosylmethionine diet defines offspring immune response via histone H3K4me3 complex and endoplasmic reticulum UPR. Cell Commun. Signal 23, 397. 10.1186/s12964-025-02386-7 40993686 PMC12462273

[B12] Liu F. Hong C. A. Gong S. Fan Z. Xiao X. Xiao Y. (2025b). Luteolin decreases fat accumulation and extends lifespan in *Caenorhabditis elegans* via DAF-16/FOXO and NHR-49/PPAR-α. J. Agric. Food Chem. 73, 30749–30760. 10.1021/acs.jafc.5c08997 41261365

[B13] Lu W. Wu Z. Zhang C. Gao T. Ling X. Xu M. (2022). Jujuboside A exhibits an antiepileptogenic effect in the rat model via protection against traumatic Epilepsy-Induced oxidative stress and inflammatory responses. Evid. Based Complement. Altern. Med. 2022, 7792791. 10.1155/2022/7792791 36118077 PMC9481365

[B14] Wang M. Wang G. Zhao M. Hou L. Ma D. Yang H. (2025). Jujuboside A in ameliorating insomnia in mice via GABAergic modulation of the PVT. J. Ethnopharmacol. 349, 119939. 10.1016/j.jep.2025.119939 40354840

[B15] Wang W. Li C. Shen L. Liu Y. Zhou J. Zheng C. (2025). Sleep aid effect and mechanism of Semen zizyphi spinosae extract enriched With jujuboside and jujubogenin in sleep-deprived zebrafish. Food Sci. Nutr. 13, e70413. 10.1002/fsn3.70413 40510790 PMC12158664

[B16] Xiao Y. Liu F. Zhao P. J. Zou C. G. Zhang K. Q. (2017). PKA/KIN-1 mediates innate immune responses to bacterial pathogens in Caenorhabditis elegans. Innate Immun. 23, 656–666. 10.1177/1753425917732822 28958206

[B17] Xiao Y. Liu F. Li S. Jiang N. Yu C. Zhu X. (2020). Metformin promotes innate immunity through a conserved PMK-1/p38 MAPK pathway. Virulence 11, 39–48. 10.1080/21505594.2019.1706305 31851866 PMC6961722

[B18] Xiao Y. Zhang H. Sheng Y. Liu F. Gao J. Liu G. (2022a). Usnic acid extends healthspan and improves the neurodegeneration diseases via mTOR/PHA-4 signaling pathway in Caenorhabditis elegans. iScience 25, 105539. 10.1016/j.isci.2022.105539 36425761 PMC9679492

[B19] Xiao Y. Liu F. Kong Q. Zhu X. Wang H. Li S. (2022b). Metformin induces S-adenosylmethionine restriction to extend the Caenorhabditis elegans healthspan through H3K4me3 modifiers. Aging Cell 21, e13567. 10.1111/acel.13567 35146893 PMC8920454

[B20] Xiao Y. Liu F. Zhu X. Li S. Meng L. Jiang N. (2023a). Dioscin integrates regulation of monosaturated fatty acid metabolism to extend the life span through XBP-1/SBP-1 dependent manner. iScience 26, 106265. 10.1016/j.isci.2023.106265 36936783 PMC10014289

[B21] Xiao Y. Zhang L. Liu Y. (2023b). Protocol for assessing the healthspan of Caenorhabditis elegans after potential anti-aging drug treatment. Star. Protoc. 4, 102285. 10.1016/j.xpro.2023.102285 37148246 PMC10193290

[B22] Xiao Y. Zhang Y. Li L. Jiang N. Yu C. Li S. (2024a). Cynaroside extends lifespan and improves the neurondegeneration diseases via insulin/IGF-1 signaling pathway in Caenorhabditis elegans. Arch. Gerontol. Geriatr. 122, 105377. 10.1016/j.archger.2024.105377 38412790

[B23] Xiao Y. Liu F. Wu Q. Zhu X. Yu C. Jiang N. (2024b). Dioscin activates endoplasmic reticulum unfolded protein response for defense against pathogenic bacteria in Caenorhabditis elegans via IRE-1/XBP-1 pathway. J. Infect. Dis. 229, 237–244. 10.1093/infdis/jiad294 37499184

[B24] Xiao Y. Hong C. a. Liu F. Shi D. Zhu X. Yu C. (2024c). Caffeic acid activates mitochondrial UPR to resist pathogen infection in Caenorhabditis elegans via the transcription factor ATFS-1. Infect. Immun. 92, e0049423. 10.1128/iai.00494-23 38294242 PMC10929418

[B25] Xiao Y. Zhou H. Cui Y. Zhu X. Li S. Yu C. (2024d). Schisandrin A enhances pathogens resistance by targeting a conserved p38 MAPK pathway. Int. Immunopharmacol. 128, 111472. 10.1016/j.intimp.2023.111472 38176342

[B26] Xiao Y. Han C. Li X. Zhu X. Li S. Jiang N. (2024e). S-Adenosylmethionine (SAM) diet promotes innate immunity via histone H3K4me3 complex. Int. Immunopharmacol. 131, 111837. 10.1016/j.intimp.2024.111837 38471365

[B27] Xiao Y. Zhang L. Zhou H. Cui Y. Chen K. Zhang H. (2025a). Berberine extends healthspan and delays neurodegenerative diseases in Caenorhabditis elegans through ROS-Dependent PMK-1/SKN-1 activation. Arch. Gerontol. Geriatr. 128, 105644. 10.1016/j.archger.2024.105644 39357500

[B28] Xiao Y. Zhang H. Li X. Han C. Liu F. (2025b). DEAD-Box RNA helicase DDX-23 mediates dietary restriction induced health span in Caenorhabditis elegans. Geroscience 47, 153–165. 10.1007/s11357-024-01434-3 39578298 PMC11872819

[B29] Xiao Y. Li L. Han C. Huang T. Ren S. Wang X. (2025c). Chlorogenic acid inhibits pseudomonas toxin pyocyanin and activates mitochondrial UPR to protect host against pathogen infection. Sci. Rep. 15, 5508. 10.1038/s41598-025-90255-1 39953205 PMC11829045

[B30] Xiao Y. Cui Y. Zhang Y. Fu W. Liu Y. Liu F. (2025d). Berberine hydrochloride enhances innate immunity to protect against pathogen infection via p38 MAPK pathway. Front. Immunol. 16, 1536143. 10.3389/fimmu.2025.1536143 40092994 PMC11906452

[B31] Xiong J. Li X. Mao F. Wang N. Liu F. Xiao Y. (2025). Ursolic acid activates mitochondrial unfolded protein response to enhance innate immunity via transcription factor ATFS-1/ATF5. J. Agric. Food Chem. 73, 29629–29637. 10.1021/acs.jafc.5c09928 41213894

[B32] Zhang Y. Shao Z. Zhai Z. Shen C. Powell-Coffman J. A. (2009). The HIF-1 hypoxia-inducible factor modulates lifespan in C. elegans. PLoS One 4, e6348. 10.1371/journal.pone.0006348 19633713 PMC2711329

[B33] Zhang M. Qian C. Zheng Z. G. Qian F. Wang Y. Thu P. M. (2018). Jujuboside A promotes Aβ clearance and ameliorates cognitive deficiency in alzheimer's disease through activating Axl/HSP90/PPARγ pathway. Theranostics 8, 4262–4278. 10.7150/thno.26164 30128052 PMC6096387

[B34] Zhang W. Cheng Q. Yin L. Liu Y. Chen L. Jiang Z. (2024). Jujuboside A through YY1/CYP2E1 signaling alleviated type 2 diabetes-associated fatty liver disease by ameliorating hepatic lipid accumulation, inflammation, and oxidative stress. Chem. Biol. Interact. 400, 111157. 10.1016/j.cbi.2024.111157 39059604

[B35] Zhang Y. Zhang H. Fu W. Xiao Y. (2025). Ginkgolide B promotes fat-lowering and lifespan in Caenorhabditis elegans via DAF-2/DAF-16 signaling pathway. J. Funct. Foods 126, 106708. 10.1016/j.jff.2025.106708

[B36] Zhang H. Xiong J. Wang Q. Song Q. Meng L. Zhang H. (2025). Chrysophanol delays aging via insulin/IGF-1 signaling pathway. Free Radic. Biol. Med. 232, 269–278. 10.1016/j.freeradbiomed.2025.03.011 40086491

[B37] Zhang Z. Che X. Feng T. Zou J. Chen G. Guo W. (2025). Jujuboside A improves insomnia by maintaining mitochondrial homeostasis in prefrontal neurons. Brain Res. Bull. 226, 111372. 10.1016/j.brainresbull.2025.111372 40334994

[B38] Zhou H. Shen Y. Dong C. Feng W. Tian Y. Xiao Y. (2025). Asperuloside promotes innate immunity via IRE-1/XBP-1 mediated unfolded protein response. Bioorg Chem. 157, 108318. 10.1016/j.bioorg.2025.108318 40024200

[B39] Zhou Y. Wei Y. Wu N. Zhang C. Wu X. Gao K. (2026). Ziziphi spinosae semen: a comprehensive review on quality marker prediction based on ethnopharmacology, phytochemistry, and pharmacology. J. Ethnopharmacol. 356, 120852. 10.1016/j.jep.2025.120852 41187837

[B40] Zhu X. Liu F. Wu Q. Li S. Ruan G. Yang J. (2022). Brevilin A enhances innate immunity and the resistance of oxidative stress in Caenorhabditis elegans via p38 MAPK pathway. Int. Immunopharmacol. 113, 109385. 10.1016/j.intimp.2022.109385 36330917

