# Supplementary material for: Jujuboside A extends healthspan and enhances resistance of oxidative stress in Caenorhabditis elegans via the transcription factor DAF-16/FOXO3A and SKN-1/Nrf2
Source: Front Pharmacol. 2026 Jun 23;17:1816985. doi: 10.3389/fphar.2026.1816985 (PMC13337699; doi:10.3389/fphar.2026.1816985)
Supplement: Supplementary file 1 [file Table1.docx]

**Table S1. Primer sequences used for quantitative RT-PCR.**

| Gene | Sequences (5’→3’) |
| --- | --- |
| *pmp-3*-F  *pmp-3*-R | TGGATTGTCATTGGCGTCG  GTTGTCGCAGAGTGGTGTTT |
| *sod-3*-F  *sod-3*-R | TCCAAGCACACTCTCCCAGAT  TCTCCACCATCCTTAGCCAAG |
| *thn-2*-F  *thn-2*-R | GCTCGCACCATCACTATCTAC  CACATCCAGTTCTTGCCCAA |
| *lys-7*-F  *lys-7*-R | ATGACTCCACAGCCCGTTT  GGCGAAGTGACCTGAATCCA |
| *dod-3-F*  *dod-3-R* | AAGCCATGTTCCCGAATGAG  GCTGCGAAAAGCAAGAAAATG |
| *hsp-12.6-F*  *hsp-12.6-R* | GTGATGGCTGACGAAGGAAC  GGGAGGAAGTTATGGGCTTC |
| *gst-4-F*  *gst-4-R* | TCGGTCAGTCAATGTCTATCAC  CGGAAAAAGAATATGAAATCTCTGTAT |
| *gst-10-F*  *gst-10-R* | ATGCTCCTTGGTCAGTTGCC  TTGCTCGTTGGATCCGTTC |
| *gcs-1-F*  *gcs-1-R* | CAGGTGAATGCGATGCTTGG  CAAGCGATGAGACCTCCGTA |
